# Radiation resistance of additively manufactured CrMoTaTiV compositionally complex alloys with enhanced mechanical properties

**DOI:** 10.1038/s41598-026-54704-9

**Published:** 2026-07-31

**Authors:** E. Aydogan, O. U. Tukac, A. Ozalp, O. El-Atwani, M. R. Chancey, H. Kim, Y. Q. Wang, B. T. Camic, S. Ozturk

**Affiliations:** 1 Department of Metallurgical and Materials Engineering, Middle East Technical University, 06800 Ankara, Turkey; 2 Reactor Materials Group, Pacific Northwest National Laboratory, Richland, WA 99354 USA; 3 School of Mechanical and Materials Engineering, University College Dublin, Dublin, D04 V1W8 Ireland; 4 Materials Science and Technology Division, Los Alamos National Laboratory, Los Alamos, NM 87545 USA; 5 Sabanci University Nanotechnology Research and Application Center, 34956 Istanbul, Turkey; 6 Middle East Technical University Central Laboratory, 06800 Ankara, Turkey

**Keywords:** Refractory compositionally complex alloys (RCCAs), Additive manufacturing (AM), Radiation resistance, Mechanical properties, Electron microscopy, Engineering, Materials science

## Abstract

In this study, a novel Cr_10_Mo_25_Ta_25_Ti_15_V_25_ refractory compositionally complex alloy (RCCA) was fabricated via laser powder directed energy deposition and subjected to irradiation at doses of up to 18 dpa at room temperature (RT) and 650 °C. Microstructural analysis revealed a dendritic BCC matrix, featuring localized enrichment of Cr, V, and Ti in the interdendritic regions, alongside a secondary FCC phase rich in Ti and O. Both compressive strength and ductility are much improved compared to the alloys produced by conventional production methods. Even at 1000 °C, the yield strength of 953 ± 70 MPa with extensive compressive ductility has been observed. Irradiation studies demonstrated that both phases remained stable, with no void formation or phase transformation. The lattice parameters of BCC matrix and FCC second phase fluctuate which was mainly attributed to local variation in composition. Loop size and density followed distinct temperature-dependent trends. At RT, limited kinetics favored loop nucleation while at 650 °C, loop coalescence was dominant. The high dislocation density inherent to additive manufacturing and sluggish diffusion in the RCCA matrix, coupled with O-rich FCC particles, contributed to the alloy’s robust radiation tolerance. These findings showcase the potential of additively manufactured RCCAs for next-generation nuclear fission and fusion applications.

**Supplementary Information:**

The online version contains supplementary material available at 10.1038/s41598-026-54704-9.

## Introduction

Next-generation advanced nuclear reactors are designed to operate under harsh conditions, such as high temperatures, high radiation doses and corrosion environments. This intensifies the need for core structural materials with outstanding mechanical strength, minimal swelling and creep, and stable phases at high temperatures^1^. Compositionally complex alloys (CCAs), also known as medium- or high-entropy alloys (M/HEAs), are reported to present unconventional deformation processes^2–4^ and defect migration pathways^5^ as a result of complex local lattice distortion^6,7^, local chemical ordering^8,9^, and structural heterogeneities^10^. Thus, CCAs present new parameters for optimization, enabling exceptional mechanical performance, as well as superior corrosion, wear, and irradiation resistance^11–15^.

It is reported that, overall, CCAs offer more radiation resistance compared to the conventional alloys^16^. For instance, Lu et al.^17^ presented that with increasing complexity in the composition, from NiFe, NiCoFe, NiCoFeCr, and NiCoFeCrMn, defect evolution is delayed after irradiation to ~ 38 dpa at 773 K. However, adding elemental complexity does not always convey better properties, especially in BCC alloys. Jia et al.^18^ examined helium (He) bubble and precipitation behavior in BCC-structured V, TiVTa, and TiVTaNb alloys. Contrary to observations in single-phase solid solutions with FCC structures, increasing compositional complexity through additional alloying elements did not suppress He bubble growth and precipitation formation in these BCC alloys. On the other hand, Kareer et al.^19^ have shown that the radiation induced hardening was much less in TiVTaZr, TiVTaCr and TiVTaNb compared to pure V after ion irradiation to ~ 3.6 dpa at 500 °C. Besides, WTaVCr and WTaVCrHf RCCAs were reported to exhibit exceptional radiation resistance compared to pure W, without showing much loop and helium bubble formation in thin film forms^14,20^. Nonetheless, further in-depth research is essential to fully grasp the irradiation behavior of BCC-structured CCAs, produced in bulk form.

Conventional techniques, including vacuum arc melting (VAM) or induction melting, are frequently utilized to produce RCCAs^12,13,21^. To enhance chemical homogeneity, these techniques entail casting and multiple remelting stages^21^. Additionally, post-treatment procedures are required to achieve desired characteristics due to slow cooling rates of the traditional casting techniques, which range from 10 to 20 K/s and result in significant phase separation^22^. On the other hand, the additive manufacturing methods provide a high cooling rate thanks to local heat input and small amount of melting, making it possible to obtain a non-equilibrium fine microstructure and, as a result, extraordinary mechanical properties^23–25^. In addition, due to the localized heat input and small amount of melting, the cooling rate is high which prevents extensive elemental segregation and promotes the solid solution^23,26^. For instance, Han et al.^27^ have reported that regardless of the segregation density of the constituent elements in CoCrFeNi-based CCA, single phase solid solution can be obtained via the directed energy deposition (DED) method. Ozalp et al.^23^ have reported a single phase Hf_5_Mo_15_Nb_35_Ta_25_Ti_20_ RCCA with a small amount of Hf-Ti oxide particle formation. On the other hand, complex and repeated thermal history and long-time exposure to laser may cause cracking and columnar grain formation due to internal stress formation. Besides, in the case of refractory CCAs, there might be unmolten powders remaining in the microstructures^23,28–30^. These drawbacks can also cause weak and anisotropic mechanical behavior^31^. Fortunately, the production parameters of the DED method can be optimized effectively, providing a straightforward way to mitigate or eliminate these drawbacks.

Prior to this study, CrMoTaTiV composition was designed and studied in detail using thermophysical parameters and thermodynamic calculations based on CALPHAD^12^. Designed composition of Cr_10_Mo_25_Ta_25_Ti_15_V_25_ provides high strength with high-temperature stability. In this study, Cr_10_Mo_25_Ta_25_Ti_15_V_25_ RCCAs have been successfully produced using the laser powder DED (LP-DED) technique. Microstructural characterizations exhibit the dendritic BCC matrix with a trace amount of FCC Ti-rich second phase. These alloys show superior strength and ductility compared to the VAM produced alloys, especially at high temperatures. Irradiation studies using 3.5 MeV Fe^2+^ have been conducted to 1.8, 6 and 18 local dpa at RT and 650 °C. High dislocation density from the additive manufacturing process, coupled with sluggish diffusion in the RCCA matrix and the presence of oxygen-rich FCC particles, collectively imparts the alloy with strong radiation tolerance. To the best of authors’ knowledge, this is the first study on a BCC CrMoTaTiV alloy showing the improved mechanical properties and radiation resistance, attributed to the unique microstructure resulting from additive manufacturing.

## Experimental procedure

### Production of the alloys

LP-DED was performed using a DMG-Mori Hybrid system with an Nd: YAG laser having a maximum power of 2500 W and a spot diameter of 3 mm. Cr (99.9 wt%), Mo (99.95 wt%), Ta (99.9 wt%), Ti (99.99 wt%), and V (99.7 wt%) powders with a particle size of 50–110 μm were mixed under an Ar atmosphere and placed in the feeding hopper. A custom-made isolation chamber was built to control the process atmosphere^23^. The chamber had an outlet to compensate for incoming carrier gas and protection gas. Protection gas was blown into the chamber through an inlet at the bottom of the isolation chamber. The chamber was equipped with an Apogee SO-421 SDI-12 oxygen sensor with a sensitivity of 10 ppm to measure the oxygen level in the isolation chamber. Before each production, the chamber was purged with Ar until the oxygen level dropped below 10 ppm. The samples were produced using a laser power of 2000 W and 400 mm/min scan speed, followed by 2-step remelting using a laser power of 2000 W and 750 mm/min scan speed after each layer. To control the stoichiometry, the amount of the powders was adjusted based on the flowability of the powders to obtain the stoichiometry of Cr_10_Mo_25_Ta_25_Ti_15_V_25_. The thin walls of 1 cm height and 3 cm length were produced on a steel substrate at room temperature under Ar atmosphere. Table 1 shows the processing parameters, including the areal energy density ($\:AED=\frac{P}{v*t}$) and linear energy density ($\:LED=\frac{P}{v}$) where *P* is the power, *V* is the scan speed and *t* is the layer thickness.

**Table 1:** Processing parameters of LP-DED.

	Deposition		Remelting	
Deposition rate (g/min)	10		–	
Power (W)	2000		2000	
Scan speed (mm/s)	6.6		12.5	
Thickness (mm)	0.9		–	
	AED (W/mm^2^)	LED ( W/mm)	AED (W/mm^2^)	LED ( W/mm)
	336.7	303.0	177.8	160.0

### Irradiations

Irradiations were conducted on 3 mm discs of Cr_10_Mo_25_Ta_25_Ti_15_V_25_ (300–400 μm in thickness) using 3.5 MeV Fe^2+^ ions in the Ion Beam Materials Laboratory at LANL. The disks were prepared by mechanical polishing followed by electropolishing using a solution of 10 vol% perchloric acid – 90% methanol at − 40 °C. These disks were irradiated to 2.5, 8.5 and 25 peak dpa corresponding to ~ 1.8, ~ 6 and ~ 18 local dpa (at 500–800 nm depth), respectively, at room temperature and 650 °C with a dose rate of ~2 × 10^−3^ dpa/sec. A defocused Fe beam was used to perform irradiations. An irradiation temperature of 650 °C has been selected as there is a secondary BCC formation at long-holding times at and above 600 °C^12^. The target chamber vacuum was kept below ~ 10^−6^ torr at both temperatures with the help of a liquid nitrogen cold trap. The damage profile was simulated with the Monte Carlo Simulation code SRIM in Kinchin-Pease base mode with a displacement energy of 40 eV, 65 eV, 90 eV, 30 eV and 57 eV for all elements Cr, Mo, Ta, Ti and V, respectively, as provided in Ref^32^. (see Supplementary Figure S1).

### Characterizations

Microstructural analyses were conducted before and after irradiation. Phase analysis of the samples was performed using X-ray diffraction (XRD) on a Bruker D8 X-ray diffractometer at a scan rate of 1°/min in the as-built condition. Samples were prepared by grinding and mechanical polishing down to 0.04 μm colloidal silica. Optical microscopy was conducted using a Huvitz HDS 5800 digital microscope and a Nikon ECLIPSE E200. In addition, scanning electron microscopy (SEM) analysis was performed using an FEI Nova Nano SEM 430 equipped with an EDAX SSDD Apollo10 EDX camera. Electron backscatter diffraction (EBSD) analysis was performed on a FEI Nova Nano SEM 430 equipped with a TSL EBSD detector at 20 kV with a step size of 1 μm. Moreover, transmission electron microscopy (TEM) analysis was performed using multiple equipment, JEOL JEM-ARM200CFEG UHR-TEM, FEI Tecnai G^2^Spirit TWIN and FEI TALOS F200S TEM before and after irradiations. Bright field TEM (BFTEM) as well as diffraction studies were performed on the as-built samples. Two-beam studies were conducted by tilting the irradiated samples from a known zone axis. Energy dispersive X-ray spectroscopy (EDX) maps were collected with a dwell time of 40–50 µs using a ThermoFisher Super X EDX detector. Before irradiation, 3 mm TEM discs were prepared with a Fischone Model 200 pit grinder and ion milled with a Gatan 691 Precision Ion Polishing System. The irradiated samples were prepared with JEOL JIB4601F with Focused Ion Beam (FIB) technique using standard lift out procedures^33^. While the coarse thinning was conducted at 30 keV energy, thinning and final cleaning steps were conducted at 16 keV and 5 keV, respectively. The lattice parameters of the phase constituents were determined using XRD and selected area electron diffractograms (SAEDs). Compression test samples with a height of 1.5 mm and a diameter of 1.5 mm were obtained from the top of the thin walls using electrical discharge machining using MAKINO U53 along the scan direction. Compression tests were conducted at room temperature on two to three samples and high temperatures on three samples for each condition, namely 800 and 1000 °C, with a strain rate of 10^−4^ s^−1^ using an Instron 5582 Universal testing machine equipped with a furnace. Hardness measurements were conducted according to ASTM E384 standard using Shimadzu HMV-2 E microhardness tester with 2 kg load and 10 s holding time. At least 15 measurements were taken from different locations in multiple builds.

## Results

### Microstructure before irradiation

**Fig. 1:**
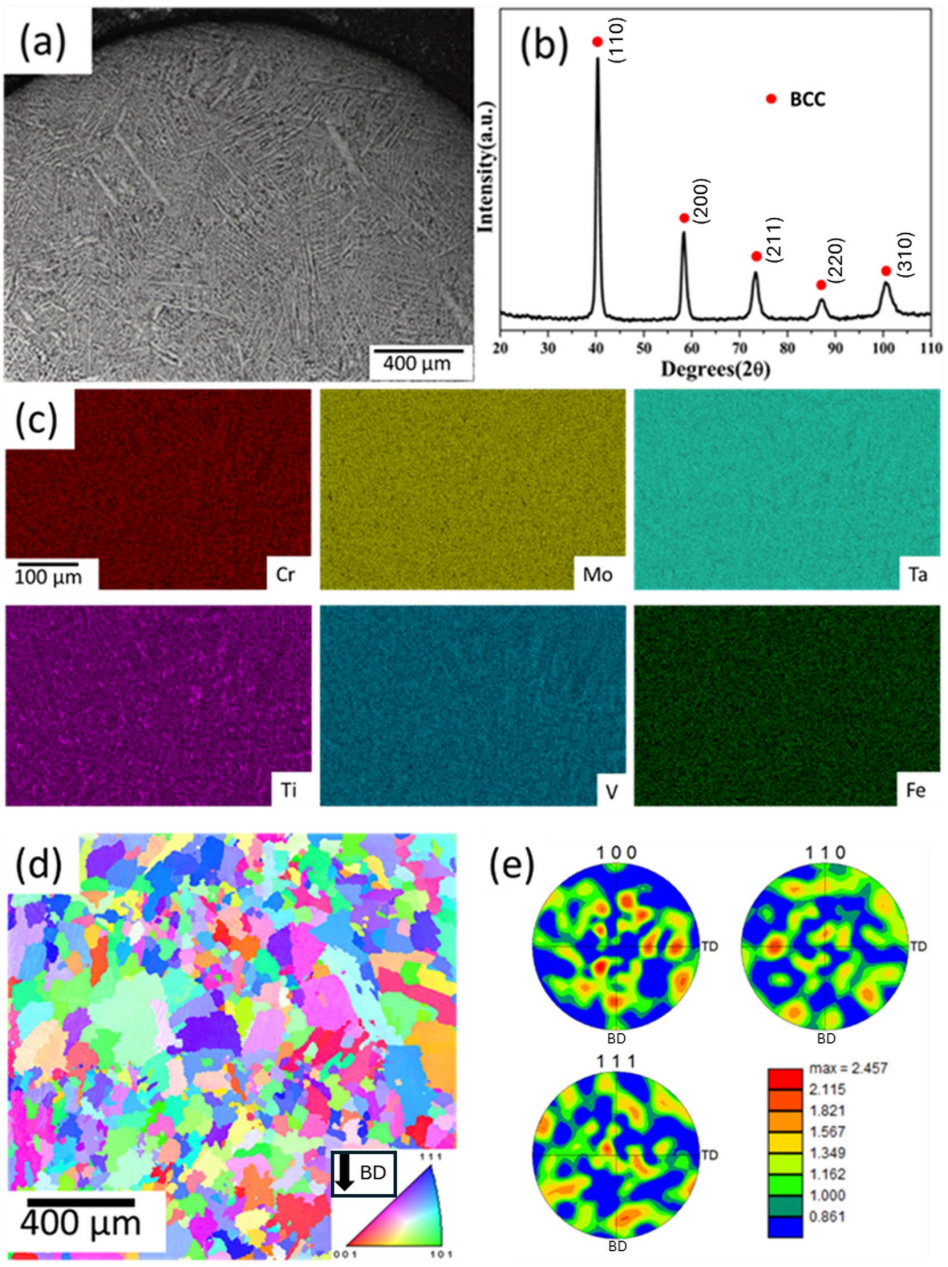
(**a**) Optical microscopy and (**b**) corresponding XRD plot of the LP-DED-produced Cr_10_Mo_25_Ta_25_Ti_15_V_25_ alloy at the upper part of the wall showing a single phase BCC. (**c**) EDX maps indicating the segregation of Cr and V in the interdendritic regions as well as the formation of Ti-rich particles. EBSD analyses showing (**d**) inverse pole figure map and (**e**) corresponding pole figures.

Figure 1a presents the optical micrograph of the LP-DED-produced Cr_10_Mo_25_Ta_25_Ti_15_V_25_ alloy showing a dendritic structure. The dendrite size remained consistent throughout the sample, due to the fast cooling of the melt pools. Moreover, the direction of dendrites in the LP-DED sample shows no preferred direction which indicates an isomorphous structure. Many fusion-based additive manufacturing methods show extreme cooling cycles going up to 10^7^ K/s^34^ which significantly confine the grain growth of the system. As previously reported in these alloys produced with vacuum arc melting, the rapid solidification structures introduce compositional inhomogeneity; V, Ti and Cr segregation and Mo and Ta depletion occur at the interdendritic regions due to melting temperature differences in the constituent elements^12^. Particularly Ti enrichment in a well-defined shape also infers the formation of Ti-rich secondary phases. On the other hand, XRD diffractogram in Fig. 1b indicates the existence of a single BCC phase with a lattice parameter of 3.16 Å. Figure 1c shows the EDX analysis conducted under SEM indicating ~ 2 at% Fe even at the upper parts of the wall (see Supplementary Table S1). While the Fe-Ta intermetallic (IM) formation was not observed at the upper part of the wall using XRD and EDX analyses, Fe inclusion was still observed. It is known that increased energy density in remelting has the potential to increase diffusion from the substrate^35^. Although IM formation was limited mostly to lower layers, Fe diffusion occurs through the built material. Indeed, even sparsely, TEM analyses on some of the samples reveal the existence of Fe-Ta intermetallic particles, as will be discussed in the following section. Many studies have shown that the diffusion of substrate material is unavoidable^35^. Compared to the VAM productions, the degree of segregation seems to be relatively minor^12^. This is attributed to the DED causing local heating and small melt pools which results in high cooling rates significantly limiting the necessary time for major segregation and maximizing the solid solution hardening^23,26^. EBSD inverse pole figure (IPF) map in Fig. 1d and corresponding pole figures in Fig. 1e indicate almost a random distribution with a low texture index. Besides, the orientation map (IPF) highlights the presence of irregular-shaped grains due to the complex thermal history of the alloys, and a relatively large grain structure with an average size of 102 ± 3 μm and bimodal grain size distribution.

**Fig. 2:**
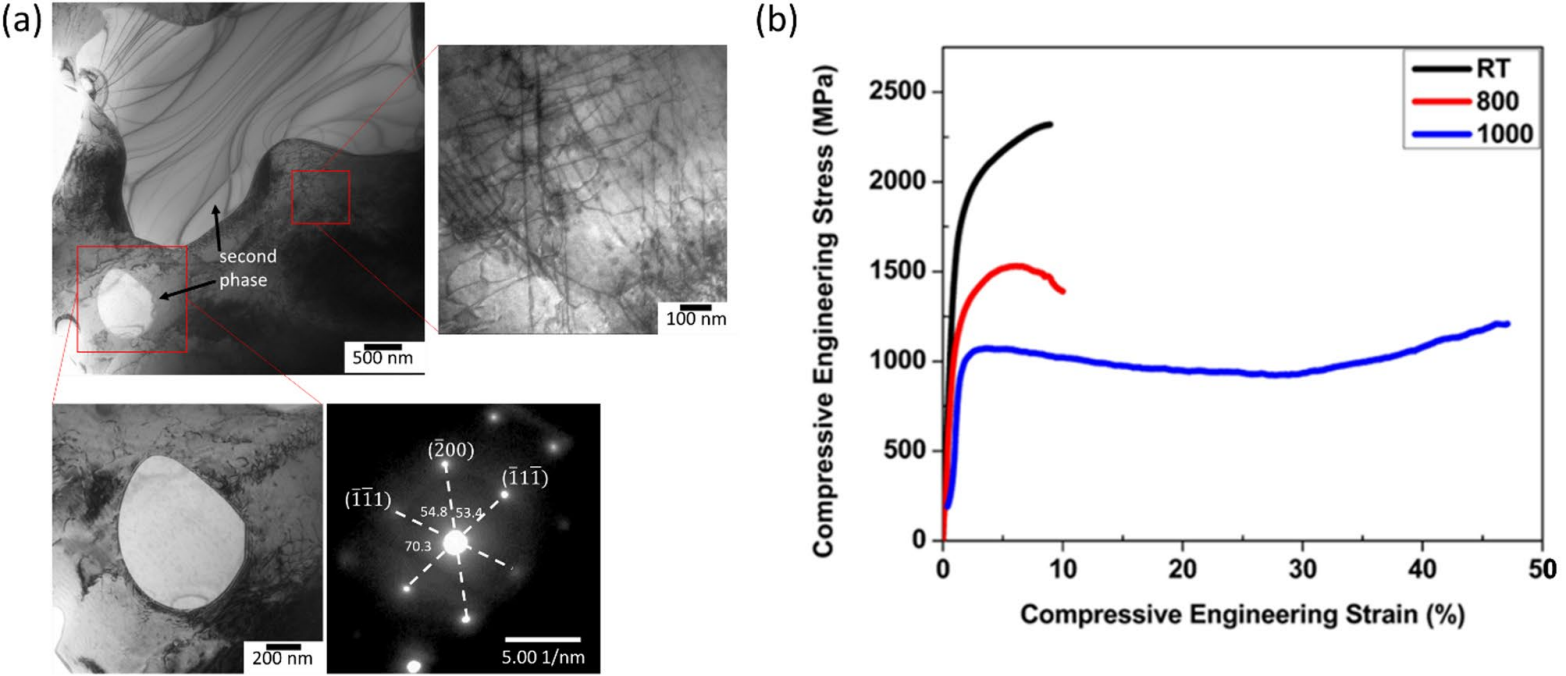
(**a**) Bright field TEM micrographs showing the dislocation structures and the second phase with selected area diffraction pattern taken along the [011] zone axis. (**b**) Compressive engineering stress-strain tests conducted at RT, 800 °C and 1000 °C.

Figure 2a shows TEM BF images of the as-built microstructure which is characterized by high dislocation density and brightly contrasted second phases. It should be noted that the volume fraction of the second phase is low; thus, it does not appear in the XRD analyses in Fig. 1b. One of the second phase particles was zoomed in to show the sharp boundary between the particle and the matrix as well as dislocation pile ups around this boundary. A representative selected area electron diffraction (SAED) pattern of a second phase has been provided exhibiting an FCC crystal structure. It should be noted that the lattice parameter of this second phase varies between 3.8 Å to 4.2 Å. Besides, these particles have been determined to be rich mainly in Titanium, Vanadium and Oxygen. Previously, the authors reported a Ti-rich FCC phase formation in the same grade produced with vacuum arc melting^12^. The lattice parameter of this phase was measured as 4.42 Å in the case of VAM-produced alloy while LP-DED production results in the formation of FCC Ti-rich phase with a smaller lattice parameter with a varying composition. This is attributed to the amount of interstitial Carbon and Oxygen in Ti as well as V, which will be discussed in detail in the following section. Furthermore, dislocations with long and wavy characteristics indicate an edge character while there are high density of loops and debris forming linearly, indicating the screw characteristic^36,37^. Similar dislocation structure was also reported in the VAM produced alloy and attributed to the rapid cooling rates due to the small size of the samples leading to significant supersaturation of the vacancies^12,38^. Even higher cooling rates (~ 10^3^-10^4^ K/s) during the LP-DED process in this study can result in similar structures.

The mechanical properties of the produced alloy were investigated using both hardness and compression tests. Hardness has been determined as 563 ± 30 HV. Figure 2b shows the compressive stress-strain behavior of the alloy. This alloy has a yield strength of 1611 ± 30 MPa at room temperature and fails at an engineering strain of 9 ± 1%. When the tests conducted at 800 °C are considered, the yield strength drops to 1100 ± 50 MPa and the plastic deformability remains similar with 10 ± 1% engineering strain. At 1000 °C, yielding occurs at 953 ± 70 MPa at which the yield stress slightly decreases due to thermal softening effects^39^. It has been observed that, at 1000 °C, the alloy has high deformability due to thermal activation of the stress carriers. The gradual increase in stress values at the higher strain side of the graph can be attributed to geometric constraints in compression mode and higher friction due to increased surface area.

### Microstructure after irradiation

Two-beam BFTEM analyses have been conducted on FIB foils. It is acknowledged that there is Ga ion damage introduced during the foil preparation. Flash electropolishing has been shown to eliminate FIB-induced damage in ferrous alloys, yet the system’s complexity makes it difficult to fine-tune both the electrolyte composition and the polishing conditions^40^. Supplementary Fig. S2 shows the BFTEM micrograph exhibiting the irradiated and unirradiated regions. To exclude the FIB damage from the Fe ion damage, the black dots due to FIB damage were characterized beyond the ion range and it is found that their size is less than 4 nm. Thus, black dots having less than 4 nm size were excluded from the statistics in the irradiated range, similar to the other studies in the literature^33,41^.

**Fig. 3:**
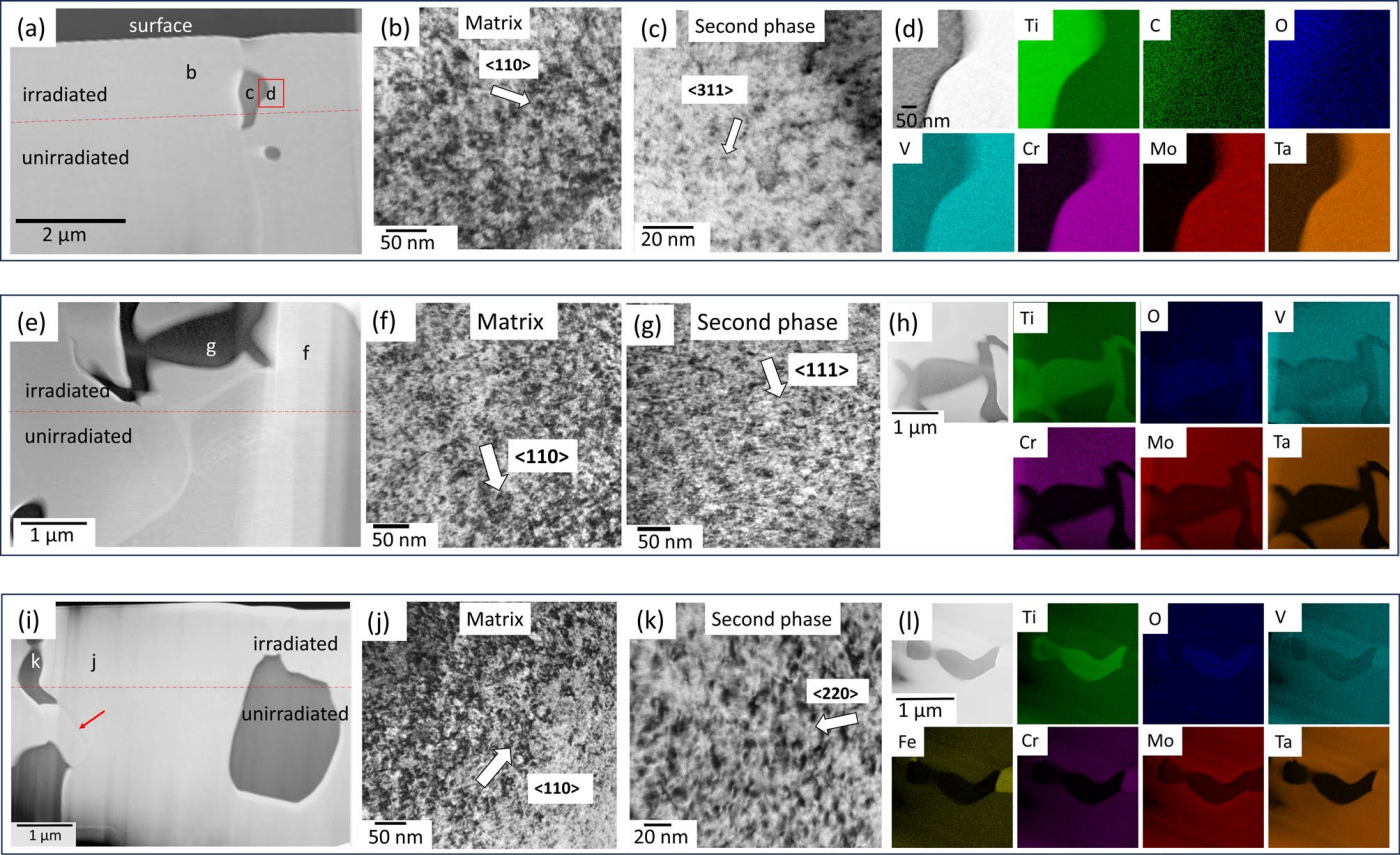
Irradiation after 1.8 dpa at RT: (**a**) HAADF image showing the distribution of the second phase; Two beam bright field TEM images of (**b**) BCC matrix (**c**) second phase; (**d**) EDX maps including the BCC matrix and the second phase. Irradiation after 6 dpa at RT: (**e**) HAADF image showing the distribution of the second phase; Two beam bright field TEM images of (**f**) BCC matrix (**g**) second phase; (**h**) EDX maps including the BCC matrix and the second phase. Irradiation after 18 dpa at RT: (**i**) HAADF image showing the distribution of the second phase; Two beam bright field TEM images of (**j**) BCC matrix (**k**) second phase; (**l**) EDX map including the BCC matrix and different second phases. Red arrow in (i) indicates the existence of another phase besides FCC Ti-rich phase.

Figure 3 shows TEM micrographs revealing the second phase distribution within the foil and the damage structure in both matrix and the second phase, as well as the EDX maps for the RT-irradiated alloys to 1.8, 6 and 18 dpa. It should be noted that the samples were titled to a g-vector from a known zone axis and the analyses have been conducted considering only the visible loops since types of the loops are difficult to determine due to their small sizes using the technique based on their shapes^33,42^. Figures 3a–d display the microstructural evolution and elemental distributions for the 1.8 dpa irradiation condition. The HAADF image in Fig. 3a reveals darker regions with distinct compositions, and two-beam analyses were performed on both the BCC matrix (g < 110>, Fig. 3b) and the FCC second phase (g < 111>, Fig. 3c). Dislocation loops in the matrix exhibit a broad size distribution up to ~ 20 nm. SAED measurements indicate a lattice parameter of 3.18 Å for the BCC matrix and 4.15 Å for the FCC second phase after 1.8 dpa at RT. EDX maps in Fig. 3d show that the second-phase regions remain rich in Ti, V, O, and C, consistent with the FCC phase present prior to irradiation. Figures 3e–h present the RT-irradiated microstructure at 6 dpa. The HAADF image in Fig. 3e again shows darker contrast associated with second-phase regions within the ion range. Two-beam analyses on the BCC matrix (Fig. 3(f)) and on the second phase (Fig. 3g) show that loop sizes in the matrix remain broadly distributed up to ~ 20 nm, while within the second-phase particles, loops are more narrowly distributed up to ~ 10 nm. EDX maps in Fig. 3h confirm that these irregularly shaped particles correspond to the FCC Ti-rich phase. The lattice parameters for this condition are measured as 3.10 Å for the BCC matrix and 3.99 Å for the FCC second phase. Figures 3i–l show the microstructure after irradiation to 18 dpa. The HAADF image in Fig. 3i indicates a region with a high fraction of darker FCC-Ti areas and the appearance of an additional phase. Two-beam imaging was conducted on the BCC matrix along g < 211> (Fig. 3j) and on the FCC phase along g < 200> (Fig. 3k). Compared to lower dpa levels, the BCC matrix exhibits smaller dislocation loops with a higher loop density, whereas the FCC second phase shows minimal change in defect size or areal density with increasing dose. SAED analyses yield lattice parameters of 3.01 Å for the BCC matrix and 4.20 Å for the FCC phase. EDX results in Fig. 3l again confirm Ti-rich second phases and additionally reveal an Fe–Ta–Cr–rich phase (highlighted in Fig. 3h), attributed to Fe diffusion from the substrate.

**Fig. 4:**
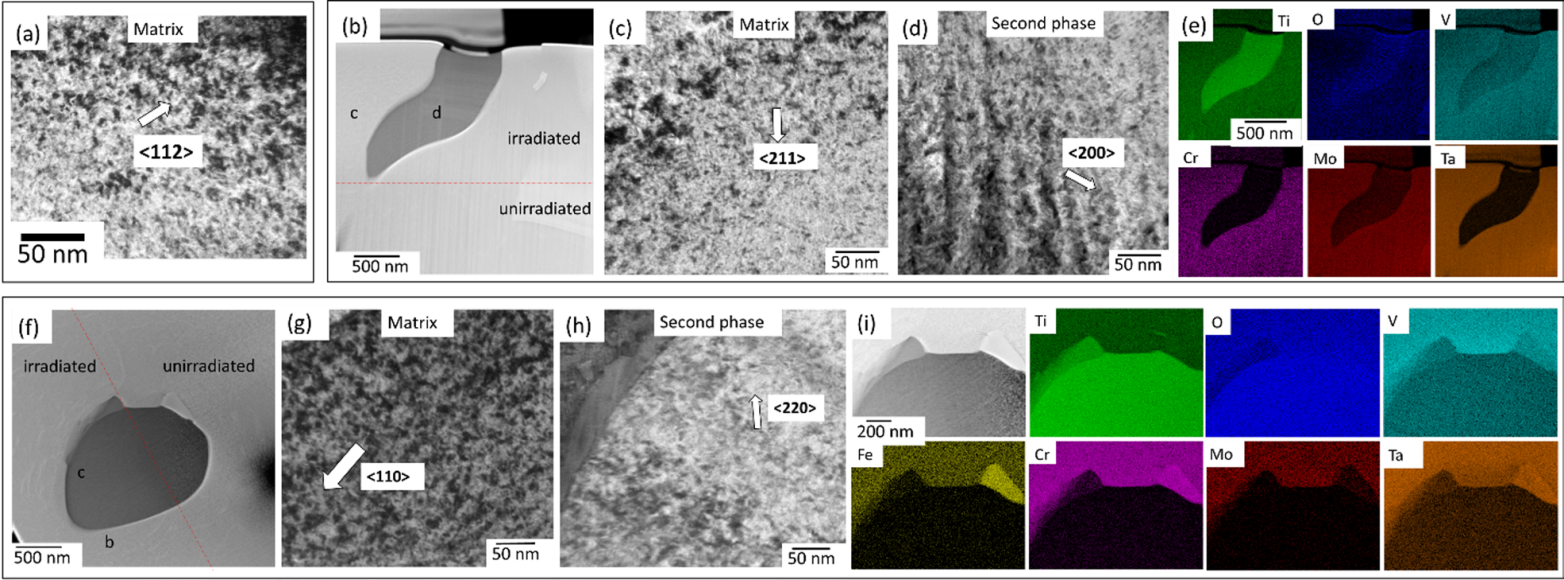
(**a**) Two beam bright field TEM images showing dislocation loop distribution in BCC matrix after 1.8 dpa irradiation at 650 °C. (**b**) HAADF image showing the distribution of the second phase; Two beam bright field TEM images of (**c**) BCC matrix (**d**) second phase; (**e**) EDX map including the BCC matrix and second phase after 6 dpa irradiation at 650 °C. (**f**) HAADF image showing the distribution of the second phase; Two beam bright field TEM images of (**g**) BCC matrix (h) second phase; (**i**) EDX map including the BCC matrix and second phases after 18 dpa irradiation at 650 °C.

High temperature irradiations were conducted at 650 °C at which there seems to be a secondary BCC forming^12^. Figure 4 shows TEM micrographs revealing the damage structure as well as the EDX maps for the alloys irradiated to 1.8, 6 and 18 dpa at 650 °C. Figure 4a presents two-beam TEM micrographs showing dislocation loops imaged along g <112> after 1.8 dpa ion irradiation at 650 °C, with loop sizes ranging from a few nanometers to ~ 20 nm. SAED analysis gives a lattice parameter of 3.06 Å for the BCC matrix. Because the fraction of the FCC second phase is low, at this dose, it was not captured in the examined FIB foils. Figures 4(b–e) show the second-phases, irradiation-induced damage, and corresponding EDX maps after irradiation at 650 °C up to 6 dpa. Two-beam BFTEM analyses in Fig. 4b were performed along g <211> for the BCC matrix and g < 200 > for the second phase, as shown in Fig. 4c and d. Some rafting is observed due to the alignment of dislocations within the FCC second phase. Relative to RT irradiations, both the BCC matrix and FCC Ti-rich phase exhibit slightly coarser loops at 650 °C. EDX maps in Fig. 4e confirm that the second phase remains Ti- and O-rich and retains an FCC structure. SAED measurements give lattice parameters of 3.07 Å for the BCC matrix and 4.07 Å for the FCC second phase. At 18 dpa, Fig. 4f shows HAADF imaging of irradiated and unirradiated regions, revealing multiple secondary phases in the matrix. Two-beam BFTEM images in Fig. 4g and h show that both the BCC matrix and FCC second phase contain slightly larger loops than at lower doses. Diffraction analyses yield lattice parameters of 3.03 Å for the BCC matrix and 4.12 Å for the FCC phase. EDX maps in Fig. 4i identify both the FCC Ti-rich phase and an Fe–Ta–Cr-rich phase, the latter attributed to Fe diffusion from the substrate.

**Fig. 5:**
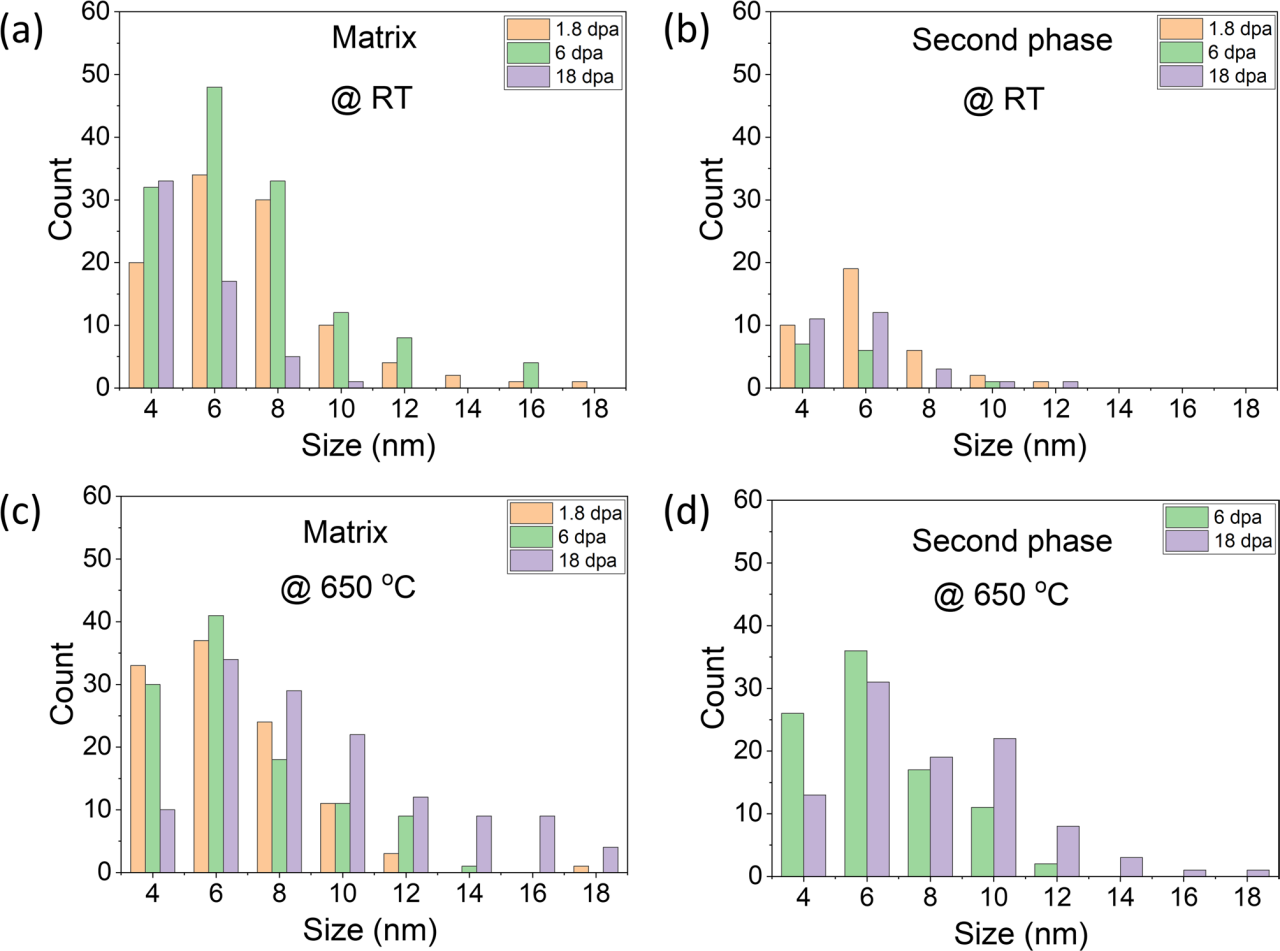
Loop size distribution after 3.5 MeV Fe^2+^ irradiations to 1.8, 6, 18 dpa (local dpa at 500–1000 nm) in (**a**) the matrix and (**b**) the second phase at RT; (**c**) the matrix and (**d**) the second phase at 650 °C.

Figures 5a and b show the loop size distribution in BCC matrix and FCC second phase after irradiations at RT to 1.8, 6 and 18 dpa. In the matrix, while the loop size distribution is broad at 1.8 dpa up to ~ 18 nm, it becomes narrower with increasing dose, inferring a decrease in the average size of the loops with increasing dose. The average loop sizes are 7.1 ± 2.6 nm, 7.1 ± 2.5 nm and 5.3 ± 1.3 nm after 1.8, 6 and 18 dpa, respectively. In FCC second phase, the distribution of the loops is narrower compared to that in BCC matrix. Overall, loop size exhibits a Gaussian distribution skewed at larger sizes. The average loop sizes are 6.5 ± 1.8 nm, 5.1 ± 1.5 nm and 5.7 ± 1.8 nm after 1.8, 6 and 18 dpa, respectively. This decrease in the loop sizes with increasing dose can be attributed to the nucleation of new small loops. Figure 5c and d exhibit the loop size distribution in BCC matrix and FCC second phase after irradiations at 650 °C to 1.8, 6 and 18 dpa. BCC matrix shows a broader size distribution with larger size at increasing doses. Even though no second phase was observed in the case of 1.8 dpa irradiation, the loop size becomes larger with broader distribution in the case of 18 dpa irradiation compared to the 6 dpa irradiation. The average loop sizes in BCC matrix are 6.6 ± 2.3 nm, 6.9 ± 2.4 nm and 9.7 ± 4.7 nm after 1.8, 6 and 18 dpa, respectively. The average loop size for the FCC second phase is, 6.4 ± 1.9 nm and 8.0 ± 2.9 nm after 6 and 18 dpa, respectively. Increasing trend in the loop size infers the coalescence and/or growth of the loops.

**Fig. 6:**
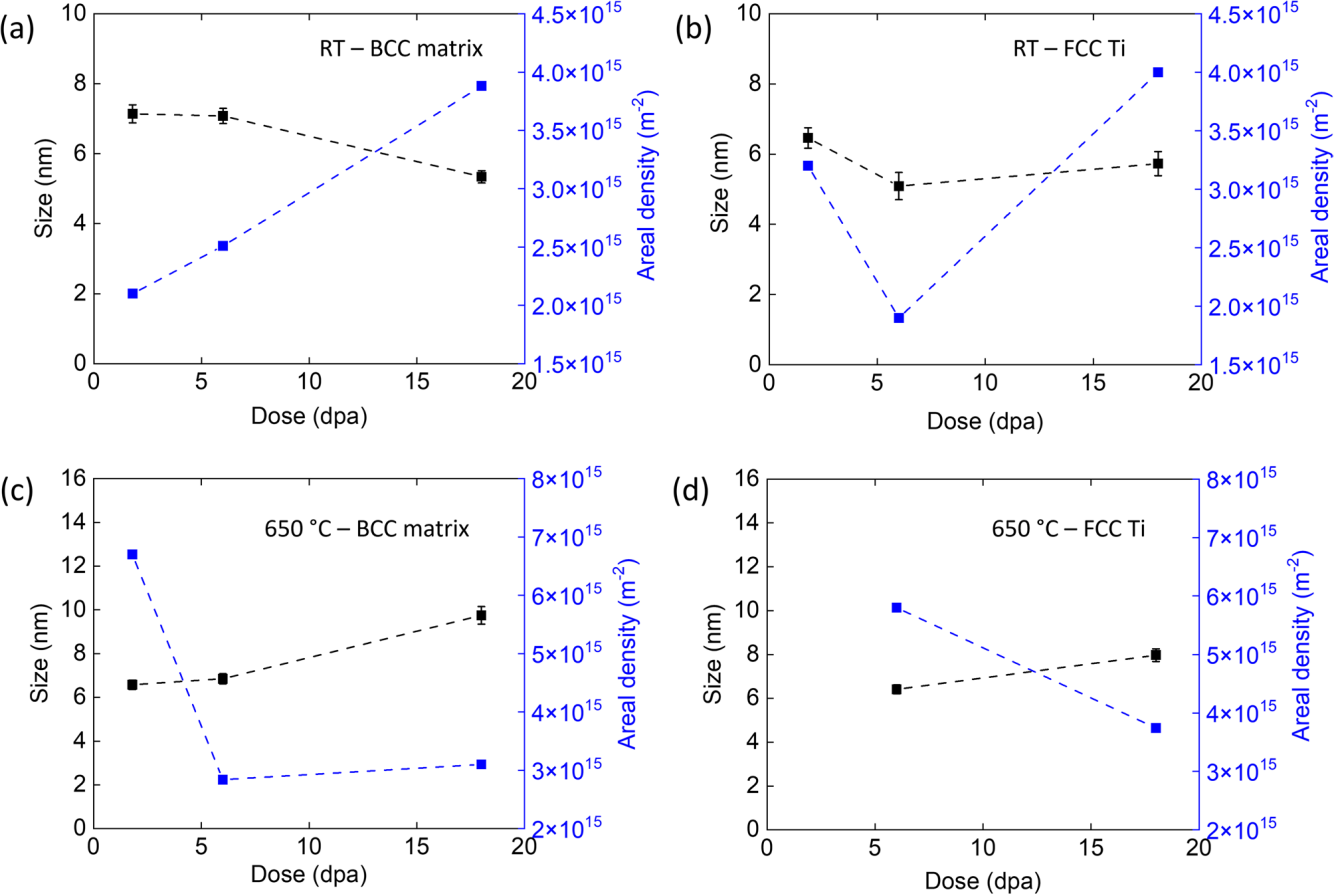
Summary of the loop statistics for; room temperature irradiation of (**a**) BCC matrix and (**b**) FCC Ti-rich phase, and 650 °C irradiation of (**c**) BCC matrix and (**d**) FCC Ti-rich phase.

Figure 6 summarizes the loop statistics for BCC matrix and FCC Ti-rich phase at RT and 650 °C. At RT, loop size decreases slightly with increasing dose while the number density increases considerably for BCC matrix (see Fig. 6a). On the other hand, initially, both size and areal density decrease while size is saturated and areal density increases after 6 dpa of irradiation at RT (Fig. 6b) for the case of FCC phase. At 650 °C, size increases slightly in the BCC matrix after 18 dpa of irradiation (Fig. 6c). Even though the loop density remains similar after 6 dpa, initial loop density is too high indicating the FIB damage. Moreover, the size of the loops increases slightly after 18 dpa of irradiation while the areal density decreases sharply in the FCC phase (Fig. 6d). At RT, the slight decrease in the size of the loops in both BCC and FCC structures with increasing dpa infers that nucleation still dominates over the growth of the loops due to the limited kinetics at RT. This is supported by the increasing trend of loop density with damage. This also means that loop size and density are not saturated even at 18 dpa, as opposed to the ferritic systems which exhibit saturation in loop size and density within a few dpa^43^. On the other hand, at 650 °C, the size of the loops tends to increase while the density decreases with damage inferring the coalescence of the loops.

## Discussion

### Mechanical properties in as-built condition: comparison with the conventional vacuum arc melting production methods

CrMoTaTiV alloy produced by LP-DED demonstrates relatively inhomogeneous microstructure with microsegragations as a result of rapid cooling and formation of Ti-rich particles as well as sparse existing Fe-Ta intermetallics. However, microsegregation is much less compared to the VAM produced alloys. Indeed, the variation in the hardness taken from different regions of a build as well as different builds found to be insignificant (see Supplementary Table S2). Moreover, this alloy exhibits a high yield strength and considerable strain hardening at room temperature, with failure at around 9% engineering strain. Increasing temperature enhances the lattice vibrations, lowering the elastic constants and activates the high temperature dislocation mechanisms, leading to decrease in the critical stress needed to activate dislocation motion. At 800 °C, failure occurs at lower strain values than 1000 °C. This behavior has been also observed in the CrMoTaTiV alloys produced by VAM and attributed to the increase in the fraction of the FCC phase at high temperatures which introduces increased amount of BCC/FCC boundary pinning effect^44^. On the other hand, at 1000 °C, after the yielding, thermal softening is seen which is followed by straining to the large values. This behavior can be attributed to combination of dynamic recrystallization and recovery, grain boundary sliding, dislocation slip and climb and stress assisted diffusion mechanisms^45^.

**Fig. 7:**
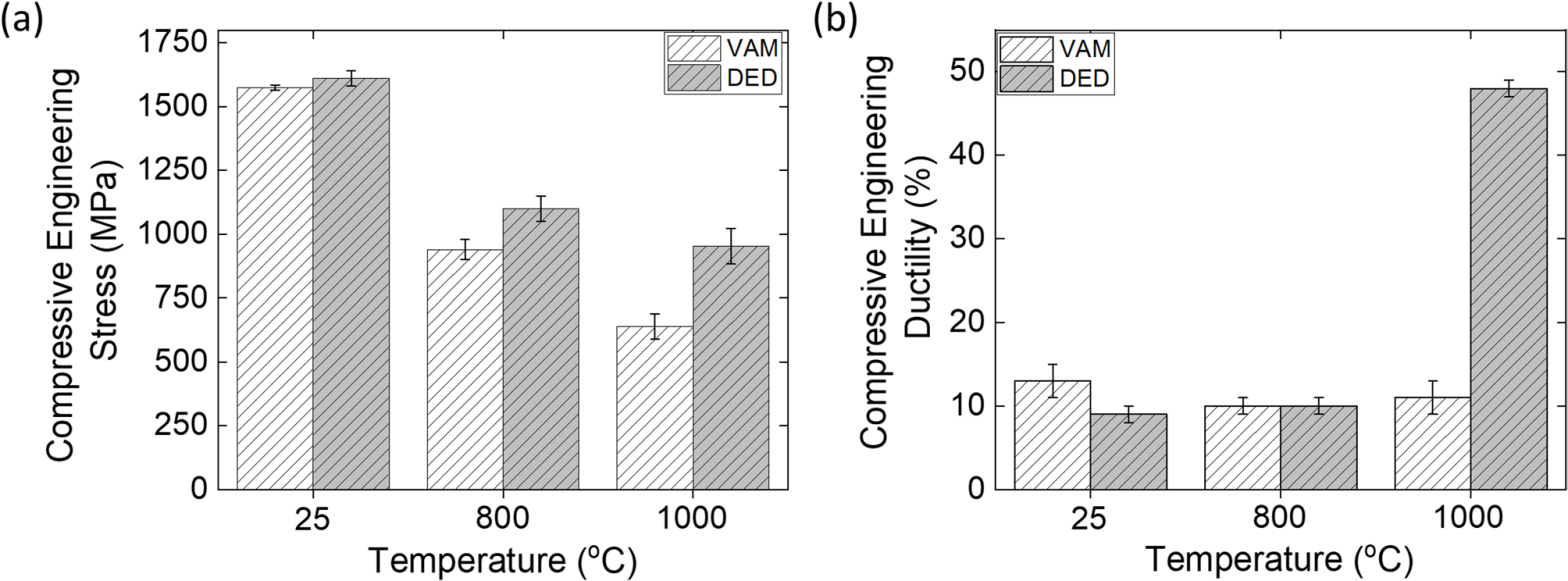
Comparison of compressive strength and ductility of LP-DED and VAM produced Cr_10_Mo_25_Ta_25_Ti_15_V_25_ alloys at RT, 800 °C and 1000 °C.

Figure 7 shows the comparison between the yield strength and ductility of the LP-DED and VAM produced alloys at RT, 800 °C and 1000 °C. The data on the yield strength and ductility of the VAM-produced alloys have been adopted from Ref^12^. The yield strengths of the LP-DED produced alloys are much higher at all temperatures. Moreover, the LP-DED processed alloys have a slightly lower ductility at RT, similar ductility values at 800 °C, and much larger ductility values at 1000 °C compared to the VAM produced alloys. In CCAs, each element atom has their unique environment and feels distortion due to different directional interactions in the local environment^46^. Generally, these distortions originate mainly from the differences in the electronic interactions between the atomic pairs in the lattice and the discrepancies arising from the differences in the sizes of the atoms sitting in the lattice^46^. The interactions between local lattice distortion and elastic field of dislocations as well as the fluctuations in these interactions impede the dislocation movement and thus increase the resistance against deformation^47^. The higher strength in LP-DED produced alloys can be attributed to the high dislocation densities as a result of DED production as well as a slight difference in their composition^48,49^. The Mo concentration in the LP-DED produced alloys is slightly higher than the ones produced by VAM (see Supplementary Table S1). Even though Mo has a similar atomic size compared with other element atoms, the shear modulus of the Mo is relatively larger among the elements and is thought to cause a higher local lattice distortion^49^. Moreover, the presence of larger lattice distortion is reported to increase the vacancy diffusion barrier, which retards the plasticity, thus, improving the strength and ductility^50^. The presence of steep thermal gradients during the processing promotes the formation of irregular-shaped grains which might result in asymmetric tensile and compressive properties^23^. Moreover, reported FCC-Ti phase may have contributions to the mechanical properties as FCC-Ti rich particles show remarkable compressive strength, and qualitatively, their number fraction is larger in the LP-DED produced alloys^51^. The existence of large incoherent FCC-Ti particles increases the pining effect of the dislocations, which is apparent from the dislocation pileups around the interface in Fig. 2.

### Radiation response in additively manufactured RCCAs

Additive manufacturing of RCCAs has been gaining much attention, however, studies on the radiation response of the bulk RCCAs are limited. While the studies on nano-structured RCCA thin films show outstanding radiation resistance, this study represents one of the first studies on the radiation response of the additively manufactured bulk RCCAs. Additively manufactured austenitic and ferritic/martensitic steels have been reported to present improved mechanical properties as well as radiation resistance. This has been attributed to the formation of dendritic/cellular structure as a result of pile up of dislocations during non-equilibrium cooling process^52–54^. Indeed, the cell boundaries have been determined to be the main defect sinks in IN-718 alloys with nano-particles^54^. In this study, the formation of dendritic structure can be seen in Fig. 1a and c. Moreover, both KAM map (Supplementary Figure S3) and TEM micrographs (Fig. 2) exhibit the presence of high dislocation density in as-built condition. Collette and King^55^ have used molecular dynamics simulations to study the influence of initial dislocation density in additively manufactured 316 L stainless steel and reported that the dislocation dense regions act as neutral sinks and reduce the probability of defect formation at first few dpa. This might delay the formation of defects. However, after multiple cascades, dislocations lose their sink efficiency and defects start to form due to irradiation-enhanced diffusion^55^. The formation of defects might be even further delayed in CCAs in which the dislocations are pinned by the lattice strains and sluggish diffusion as a result of lattice distortion.

While in ferritic and austenitic alloys the dislocation loop density can decrease by several orders of magnitude with increasing irradiation temperature, largely due to the enhanced diffusion of interstitials and vacancies, this decline is much less pronounced in FCC CCAs^56^. The relatively minor changes in loop density and size in CCAs at higher temperatures are attributed to sluggish diffusion kinetics, and in CCAs specifically, smaller loop sizes have been reported due to the enhanced recombination of point defects rather than loop growth. Lu et al.^17^ demonstrated this effect by preparing a series of single-phase solid-solution alloys (NiFe, NiCoFe, NiCoFeCr, and NiCoFeCrMn), which, after irradiation to 38 ± 5 dpa at 773 K using 3 MeV Ni^2+^ ions, exhibited an increase in loop number density and a decrease in loop size with increasing alloying species, indicating that additional alloying elements delay loop evolution. Furthermore, with higher irradiation doses, such as increasing from 50 dpa to 100 dpa in CCA coatings, the loop size continues to grow while the number density decreases. This observation suggests that at 50 dpa the dislocation loops have not reached saturation; as the dose increases, the loops preferentially absorb more point defects, and merging of loops becomes more probable, resulting in larger loops and a simultaneous decrease in their overall number density^57^. In this study, the size of the loops is smaller, and their density is higher compared to the conventional alloys, especially at low temperatures. For instance, the loop size and number density at the two-beam conditions are ~ 5 nm and ~ 3.9 × 10^22^ m^−3^ after 18 dpa at RT while the loop size and total number density (including < 111 > and < 100 > loops) are ~ 11 nm and 3-4 × 10^22^ m^−3^, respectively in FeCrAl BCC alloys after 16 dpa at 300 °C^33^. At RT, with dose, size decreases slightly while the density increases. Local chemical ordering and increased distortion in compositionally complex alloys make the diffusion of vacancies and interstitials localized, and thus resulting in formation of small loops, rather than growth and coalescence^1^. Mei et al. reported the predominance of < 100 > type sessile dislocation loops after heavy ion irradiations on TaTiV CCAs at RT^58^. In this study, immobile < 100 > loops might inhibit the coalescence while increasing dose forms the discrete loops. At high temperatures, the size of the loops is slightly coarser and increases with dose while the loop density decreases. This indicates the coalescence of the loops. At the temperatures of (0.3–0.6)T_m_, where T_m_ is the melting temperature, the vacancies start clustering and form voids^59^. However, in this study, even at high temperatures, there are no voids observed. This can be attributed to the recombination of the Frenkel pairs due to the migration energy barrier of the point defects even at relatively high temperatures. A similar trend has been reported in other BCC CCAs. For instance, Wang et al. studied the radiation response of WTaNbMo alloys using 2.7 MeV Si^2+^ ions to a peak damage of 50 dpa at 450, 550 and 600 °C. They reported that the average size increased slightly with increasing temperature while the density decreased slightly^60^.

Ti-rich FCC second phase shows a similar behavior to the BCC matrix. Even though the void formation has been reported at relatively low doses in FCC CCAs, such as Cantor alloys, there are no voids detected in the FCC second phase in this study^17^. It has been reported that existence of interstitial oxygen can introduce local chemical ordering and increase local lattice distortion further^1^. The local lattice distortion as a result of the local chemical environment affects the migration energies of vacancies and interstitials. If the migration energies for vacancies and interstitials become closer, recombination of the defects occur^61^. The presence of interstitial oxygen also enhances the mechanical performance of medium- and high-entropy alloys by immobilizing and multiplying dislocations, which promotes a more uniform distribution of these moving defects^1,13^.

CrMoTaTiV alloy produced by LP-DED maintains stability under irradiation. In other words, the pre-existing FCC phase remains stable while there is no extra phase formation after irradiation. It should be noted that the FCC phase which is composed of mainly Ti and O as well as varying amounts of Ta and V (see Supplementary Table S3) has varying composition in both unirradiated and irradiated conditions. Moreover, microsegregation as a result of rapid cooling during LP-DED seems to be reduced after irradiation, as evidenced by the EDX maps in Figs. 3 and 4, which does not show any indication of segregation. While this can be attributed to ballistic mixing due to irradiation, it requires further detailed investigation.

The lattice parameters of both matrix and second phase fluctuate from 3.01 to 3.18 Å and 3.99 to 4.20 Å, respectively after irradiation at RT and 650 °C. Besides the change in elemental contents of these phases partitioning between matrix and the second phase as a result of irradiation, formation of defects affects the lattice parameters. Increase in the lattice parameter in conventional alloys and carbides have been reported commonly after irradiation^62^. It has been reported that while the point defects and small defect clusters increase the strain and lattice parameter, larger defect clusters can decrease the lattice parameter^63^. For instance, Helium irradiations on some compositionally complex alloys such as FeCrVMn, NiCoFeCr and NiCoFeCrMn CCAs resulted in an increase in the lattice parameters, similar to conventional alloys^63,64^. However, Lu et al.^65^ reported that lattice parameters in Ti_2_ZrHfV_0.5_Mo_0.2_ system decreases after irradiation. This has been attributed to relaxation in the lattice distortion as opposed to the conventional alloys and some CCAs^65^. Thus, due to the complexity of the microstructure in this study, mechanism of the fluctuation of lattice parameters is not clear.

## Conclusions

In this study, a novel grade of Cr_10_Mo_25_Ta_25_Ti_15_V_25_ RCCA previously developed by the authors was produced by additive manufacturing techniques, and irradiation studies were conducted up to 18 dpa at RT and 650 °C. Microstructure of the alloys produced by LP-DED is characterized by a dendritic structure with Cr, V and Ti enrichment at the interdendritic regions. Even though the XRD analysis indicates a single-phase BCC phase with 3.16 Å lattice parameter, EDX maps and detailed TEM analyses exhibit the presence of the FCC second phase which is rich in Ti and O with varying amounts of V and Ta and varying lattice parameter depending on the composition. Both the EBSD and TEM analyses exhibit the high density of dislocations, typical of additively manufactured materials. The mechanical behavior of the produced alloy was characterized through compression tests and compared with the mechanical properties of the alloys produced by VAM. At room temperature, the alloy exhibits a yield strength of about 1600 MPa and fails at an engineering strain of approximately 9%. When tested at 800 °C, its yield strength decreases to nearly 1100 MPa while retaining a similar plastic deformability of around 10%. Even at 1000 °C, the alloy begins to yield at roughly 950 MPa, after which its yield stress gradually diminishes due to thermal softening effects. The improvement of the yield strength compared to the VAM produced alloys was attributed to the higher dislocation density and higher fraction of Ti-rich FCC second phase in LP-DED produced alloys.

Irradiation studies on the LP-DED produced alloys have shown that both BCC matrix and FCC second phase exhibit similar radiation response at RT and 650 °C. The material is stable under irradiation; there is no phase transformation. While there is no void formation at both temperatures, high density of loops with small sizes are observed. The lattice parameters of both BCC matrix and FCC second phase fluctuate with irradiation both at RT and 650 °C. This has been attributed to mainly compositional fluctuation with possible contributions from loop damage and relaxation in the lattice distortion. The size of the loops decreases slightly while the density increases with increasing dose at RT inferring that the nucleation is dominant over the growth due to the limited kinetics at this temperature. At 650 °C, the size of the loops increases with dose while the density decreases, indicating a coalescence of the loops. Still, due to the dominance of the recombination, no voids were observed at this temperature. Moreover, the existence of the high dislocation density in highly distorted lattice structures may be effective on delaying the radiation damage to higher doses. Overall smaller loop sizes with larger number density compared to conventional alloys has been attributed to the high dislocation density as a result of additive manufacturing, sluggish diffusion in the matrix and O-rich concentrated second phase particles. This study exhibits the promise of the additively manufactured refractory compositionally complex alloys for the next generation fission and fusion applications.

## Supplementary Information

Below is the link to the electronic supplementary material.

Supplementary Material 1

## Data Availability

Data will be made available on request.

## References

[1] Su, Z. et al. Interstitial oxygen solutes promote atomic-scale heterogeneities to achieve superior irradiation tolerance in body-centered cubic multi-principal element alloys. *J. Mater. Sci. Technol.* **227**, 142–154 (2025).

[2] Ding, Q. et al. Tuning element distribution, structure and properties by composition in high-entropy alloys. *Nature* **574** (7777), 223–227 (2019). 31597974 10.1038/s41586-019-1617-1

[3] Wang, H. et al. Deformation-induced crystalline-to-amorphous phase transformation in a CrMnFeCoNi high-entropy alloy. *Sci. Adv.* **7** (14), eabe3105 (2021). 33789894 10.1126/sciadv.abe3105 PMC8011962

[4] Xu, B. et al. Harnessing instability for work hardening in multi-principal element alloys. *Nat. Mater.* **23** (6), 755–761 (2024). 38605195 10.1038/s41563-024-01871-7 PMC11150159

[5] Wang, F. et al. Multiplicity of dislocation pathways in a refractory multiprincipal element alloy. *Science* **370** (6512), 95–101 (2020). 33004516 10.1126/science.aba3722

[6] Utt, D. et al. The origin of jerky dislocation motion in high-entropy alloys. *Nat. Commun.* **13** (1), 4777 (2022). 35970838 10.1038/s41467-022-32134-1 PMC9378647

[7] Yin, S., Ding, J., Asta, M. & Ritchie, R. O. Ab initio modeling of the energy landscape for screw dislocations in body-centered cubic high-entropy alloys. *npj Comput. Mater.* **6** (1), 110 (2020).

[8] Chen, X. et al. Direct observation of chemical short-range order in a medium-entropy alloy. *Nature* **592** (7856), 712–716 (2021). 33911276 10.1038/s41586-021-03428-z

[9] He, Z. et al. Interstitial-driven local chemical order enables ultrastrong face-centered cubic multicomponent alloys. *Acta Mater.* **243**, 118495 (2023).

[10] Ma, E. & Wu, X. Tailoring heterogeneities in high-entropy alloys to promote strength–ductility synergy. *Nat. Commun.* **10** (1), 5623 (2019). 31819051 10.1038/s41467-019-13311-1 PMC6901531

[11] Cook, D. H. et al. Kink bands promote exceptional fracture resistance in a NbTaTiHf refractory medium-entropy alloy. *Science* **384** (6692), 178–184 (2024). 38603511 10.1126/science.adn2428

[12] Tukac, O. U., Ozalp, A. & Aydogan, E. Development and thermal stability of Cr10Mo25Ta25Ti15V25 refractory high entropy alloys. *J. Alloys Compd.* **930**, 167386 (2023).

[13] Iroc, L. K. et al. Design of oxygen-doped TiZrHfNbTa refractory high entropy alloys with enhanced strength and ductility. *Mater. Design*. **223**, 111239 (2022).

[14] El Atwani, O. et al. A quinary WTaCrVHf nanocrystalline refractory high-entropy alloy withholding extreme irradiation environments. *Nat. Commun.* **14** (1), 2516 (2023). 37130885 10.1038/s41467-023-38000-y PMC10154406

[15] Zhang, Z., Han, E. H. & Xiang, C. Corrosion behaviors of Mo0.5NbTiVCr0.25 and Mo0.5NbTiV0.5Zr0.25 multi principal element alloys in high temperature borated and lithiated water. *Corros. Sci.* **206**, 110514 (2022).

[16] Cheng, Z. et al. Irradiation effects in high-entropy alloys and their applications. *J. Alloys Compd.* **930**, 166768 (2023).

[17] Lu, C. et al. Radiation-induced segregation on defect clusters in single-phase concentrated solid-solution alloys. *Acta Mater.* **127**, 98–107 (2017).

[18] Jia, N. et al. Helium bubble formation in refractory single-phase concentrated solid solution alloys under MeV He ion irradiation. *J. Nucl. Mater.* **550**, 152937 (2021).

[19] Kareer, A. et al. Short communication: ‘Low activation, refractory, high entropy alloys for nuclear applications’. *J. Nucl. Mater.* **526**, 151744 (2019).

[20] El-Atwani, O. et al. Outstanding radiation resistance of tungsten-based high-entropy alloys. *Sci. Adv.* **5** (3), eaav2002 (2019). 30838329 10.1126/sciadv.aav2002 PMC6397024

[21] Gou, S. et al. Additive manufacturing of ductile refractory high-entropy alloys via phase engineering. *Acta Mater.* **248**, 118781 (2023).

[22] Kim, J., Wakai, A. & Moridi, A. Materials and manufacturing renaissance: Additive manufacturing of high-entropy alloys. *J. Mater. Res.* **35** (15), 1963–1983 (2020).

[23] Ozalp, A., Okuyucu, C., Koc, B., El-Atwani, O. & Aydogan, E. Development and directed energy deposition of high strength Hf5Mo15Nb35Ta25Ti20 refractory high entropy alloys. *Mater. Charact.* **209**, 113679 (2024).

[24] Zhu, Y. et al. Ultrastrong nanotwinned titanium alloys through additive manufacturing. *Nat. Mater.* **21** (11), 1258–1262 (2022). 36109672 10.1038/s41563-022-01359-2

[25] Ren, J. et al. Strong yet ductile nanolamellar high-entropy alloys by additive manufacturing. *Nature* **608** (7921), 62–68 (2022). 35922499 10.1038/s41586-022-04914-8

[26] Laurent-Brocq, M. et al. Insights into the phase diagram of the CrMnFeCoNi high entropy alloy. *Acta Mater.* **88**, 355–365 (2015).

[27] Han, C. et al. Recent advances on high-entropy alloys for 3D printing. *Adv. Mater.* **32** (26), 1903855 (2020). 10.1002/adma.201903855 32431005

[28] Malatji, N., Lengopeng, T., Pityana, S. & Popoola, A. P. I. Microstructural, mechanical and electrochemical properties of AlCrFeCuNiWx high entropy alloys. *J. Mater. Res. Technol.* **11**, 1594–1603 (2021).

[29] Melia, M. A. et al. High-throughput additive manufacturing and characterization of refractory high entropy alloys. *Appl. Mater. Today*. **19**, 100560 (2020).

[30] Pegues, J. W. et al. In situ synchrotron X-ray imaging and mechanical properties characterization of additively manufactured high-entropy alloy composites. *J. Alloys Compd.* **876**, 159505 (2021).

[31] Guo, M. et al. Selective laser melting additive manufacturing of pure tungsten: Role of volumetric energy density on densification, microstructure and mechanical properties. *Int. J. Refract. Met. Hard Mater.* **84**, 105025 (2019).

[32] Konobeyev, A. Y., Fischer, U., Korovin, Y. A. & Simakov, S. P. Evaluation of effective threshold displacement energies and other data required for the calculation of advanced atomic displacement cross-sections. *Nuclear Energy Technol.* **3** (3), 169–175 (2017).

[33] Aydogan, E. et al. Microstructure and mechanical properties of FeCrAl alloys under heavy ion irradiations. *J. Nucl. Mater.* **503**, 250–262 (2018).

[34] Wang, P. et al. Additively manufactured CoCrFeNiMn high-entropy alloy via pre-alloyed powder. *Mater. Design*. **168**, 107576 (2019).

[35] Dobbelstein, H., Gurevich, E. L., George, E. P., Ostendorf, A. & Laplanche, G. Laser metal deposition of compositionally graded TiZrNbTa refractory high-entropy alloys using elemental powder blends. *Additive Manuf.* **25**, 252–262 (2019).

[36] Wang, R. et al. Achieving high strength and ductility in nitrogen-doped refractory high-entropy alloys. *Mater. Design*. **213**, 110356 (2022).

[37] Wu, M. et al. Dislocation glide and mechanical twinning in a ductile VNbTi medium entropy alloy. *J. Mater. Sci. Technol.* **110**, 210–215 (2022).

[38] Fischer, F. D., Svoboda, J., Appel, F. & Kozeschnik, E. Modeling of excess vacancy annihilation at different types of sinks. *Acta Mater.* **59** (9), 3463–3472 (2011).

[39] Senkov, O. N., Wilks, G. B., Scott, J. M. & Miracle, D. B. Mechanical properties of Nb25Mo25Ta25W25 and V20Nb20Mo20Ta20W20 refractory high entropy alloys. *Intermetallics* **19** (5), 698–706 (2011).

[40] Edwards, D. J. et al. Understanding and removing FIB artifacts in metallic TEM samples using flash electropolishing. *J. Nucl. Mater.* **606**, 155618 (2025).

[41] Aitkaliyeva, A., Madden, J. W., Miller, B. D., Cole, J. I. & Gan, J. Comparison of preparation techniques for nuclear materials for transmission electron microscopy (TEM). *J. Nucl. Mater.* **459**, 241–246 (2015).

[42] Aydogan, E. et al. Response of 14YWT alloys under neutron irradiation: A complementary study on microstructure and mechanical properties. *Acta Mater.* **167**, 181–196 (2019).

[43] Aydogan, E., El-Atwani, O., Li, M. & Maloy, S. A. In-situ observation of nano-oxide and defect evolution in 14YWT alloys. *Mater. Charact.* **170**, 110686 (2020).

[44] Li, Z. et al. Strength-ductility synergy of Al-bearing low-density refractory TiNbMo0.5AlX medium entropy alloys via precipitation. *Mater. Sci. Engineering: A*. **844**, 143072 (2022).

[45] Alabort, E., Kontis, P., Barba, D., Dragnevski, K. & Reed, R. C. On the mechanisms of superplasticity in Ti–6Al–4V. *Acta Mater.* **105**, 449–463 (2016).

[46] Song, H. et al. Local lattice distortion in high-entropy alloys. *Phys. Rev. Mater.* **1** (2), 023404 (2017).

[47] Yin, S. et al. Atomistic simulations of dislocation mobility in refractory high-entropy alloys and the effect of chemical short-range order. *Nat. Commun.* **12** (1), 4873 (2021). 34381027 10.1038/s41467-021-25134-0 PMC8357793

[48] Li, S. H., Zhao, Y., Kumar, P. & Ramamurty, U. Effect of initial dislocation density on the plastic deformation response of 316L stainless steel manufactured by directed energy deposition. *Mater. Sci. Engineering: A*. **851**, 143591 (2022).

[49] Li, J., Yamanaka, K. & Chiba, A. Significant lattice-distortion effect on compressive deformation in Mo-added CoCrFeNi-based high-entropy alloys. *Mater. Sci. Engineering: A*. **830**, 142295 (2022).

[50] Roy, A., Singh, P., Balasubramanian, G. & Johnson, D. D. Vacancy formation energies and migration barriers in multi-principal element alloys. *Acta Mater.* **226**, 117611 (2022).

[51] Jiang, S. et al. Interstitial carbon induced FCC-Ti exhibiting ultrahigh strength in a Ti37Nb28Mo28-C7 complex concentrated alloy. *Acta Mater.* **203**, 116456 (2021).

[52] Wang, Y. M. et al. Additively manufactured hierarchical stainless steels with high strength and ductility. *Nat. Mater.* **17** (1), 63–71 (2018). 29115290 10.1038/nmat5021

[53] Li, S. et al. Evolution of cellular dislocation structures and defects in additively manufactured austenitic stainless steel under ion irradiation. *Scripta Mater.* **178**, 245–250 (2020).

[54] Aydogan, E. et al. In-situ radiation response of additively manufactured modified Inconel 718 alloys. *Additive Manuf.* **51**, 102601 (2022).

[55] Collette, R. & King, J. Molecular dynamics simulations of radiation cascade evolution near cellular dislocation structures in additively manufactured stainless steels. *J. Nucl. Mater.* **549**, 152872 (2021).

[56] Kumar, N. A. P. K., Li, C., Leonard, K. J., Bei, H. & Zinkle, S. J. Microstructural stability and mechanical behavior of FeNiMnCr high entropy alloy under ion irradiation. *Acta Mater.* **113**, 230–244 (2016).

[57] Luo, H. et al. Dislocation loops, segregation and hardening induced by high-dose ion irradiation of NbMoTaW and VCrTaW high-entropy alloy coatings on the T91 substrate. *Surf. Coat. Technol.* **473**, 130019 (2023).

[58] Mei, L. et al. Distinct ion irradiation response in VTaTi medium entropy alloy from elemental metal V. *Scripta Mater.* **223**, 115070 (2023).

[59] Hussain, A., Dhaka, R. S., Ryu, H. J., Sharma, S. K. & Kulriya, P. K. A critical review on temperature dependent irradiation response of high entropy alloys. *J. Alloys Compd.* **948**, 169624 (2023).

[60] Wang, Z. et al. High-temperature and high-dose irradiation study on the NbMoTaW high-entropy alloy coatings. *Appl. Surf. Sci.* **690**, 162658 (2025).

[61] Zhao, S. Defect properties in a VTaCrW equiatomic high entropy alloy (HEA) with the body centered cubic (bcc) structure. *J. Mater. Sci. Technol.* **44**, 133–139 (2020).

[62] Zhu, Y. et al. Microstructural damage evolution of (WTiVNbTa)C5 high-entropy carbide ceramics induced by self-ions irradiation. *J. Eur. Ceram. Soc.* **42** (6), 2567–2576 (2022).

[63] Tong, Y. et al. Evolution of local lattice distortion under irradiation in medium- and high-entropy alloys. *Materialia* **2**, 73–81 (2018).

[64] Zhang, H. et al. Effects of He-ion irradiation on the microstructures and mechanical properties of the novel Co-free VxCrFeMnNiy high-entropy alloys. *J. Nucl. Mater.* **572**, 154074 (2022).

[65] Lu, Y. et al. A promising new class of irradiation tolerant materials: Ti2ZrHfV0.5Mo0.2 high-entropy alloy. *J. Mater. Sci. Technol.* **35** (3), 369–373 (2019).

