# Supplementary material for: Radiation resistance of additively manufactured CrMoTaTiV compositionally complex alloys with enhanced mechanical properties
Source: Sci Rep. 2026 Jul 31;16:23700. doi: 10.1038/s41598-026-54704-9 (PMC13427738; doi:10.1038/s41598-026-54704-9)
Supplement: Supplementary file 1 — Supplementary Material 1 [file 41598_2026_54704_MOESM1_ESM.docx]

**Supplementary Material**


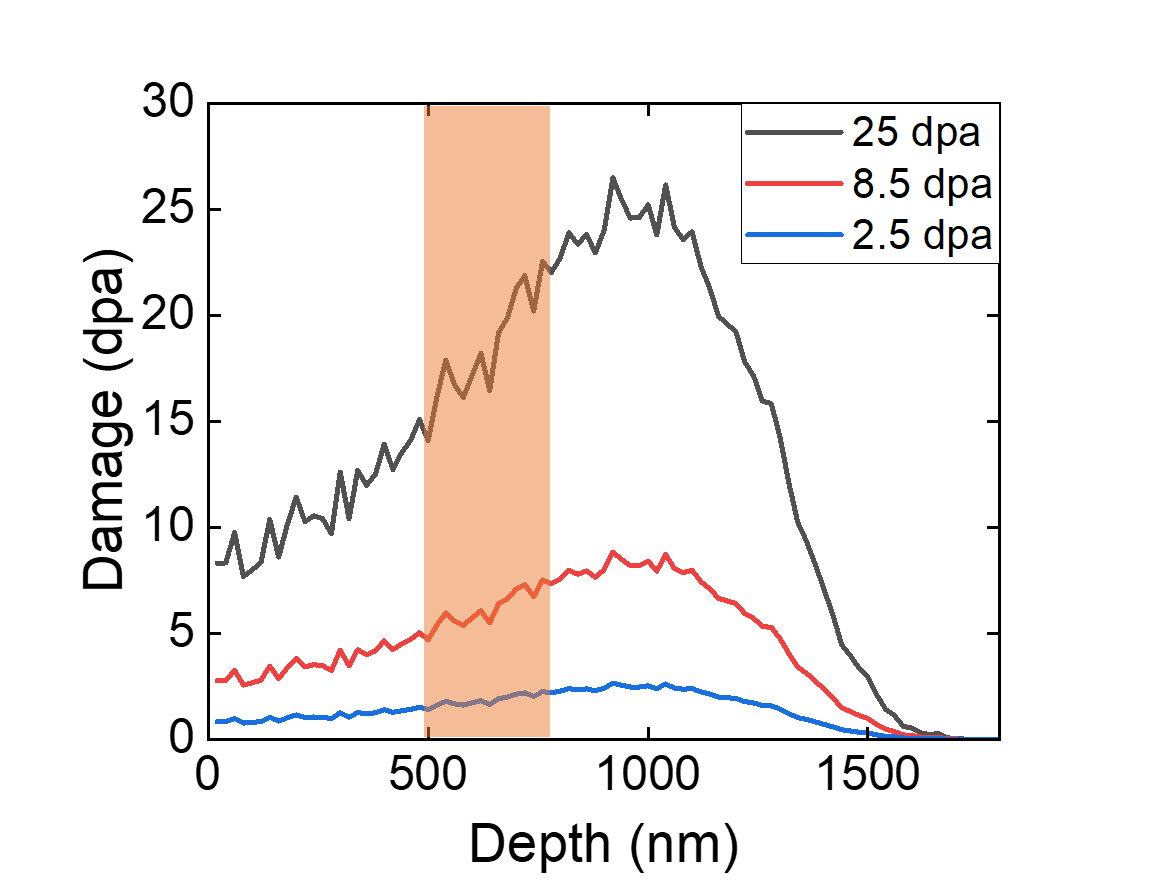


**Figure S1.** SRIM calculated damage profile for Cr_10_Mo_25_Ta_25_Ti_15_V_25_ using 3.5 MeV Fe^2+^. The shaded area corresponds to the depth of analyses (500-800 nm).


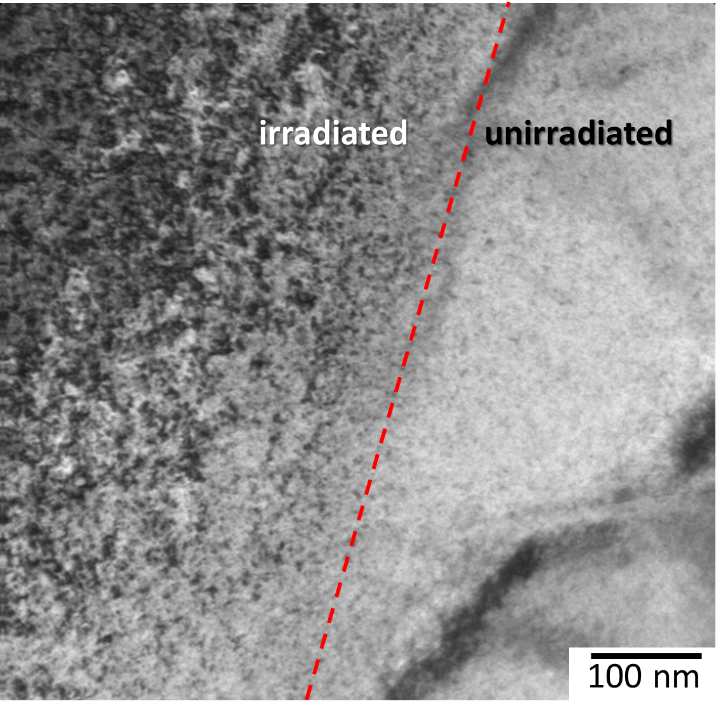


**Figure S2.** Representative BFTEM image showing the damage in irradiated and unirradiated regions on the sample irradiated to 30 local dpa at RT.


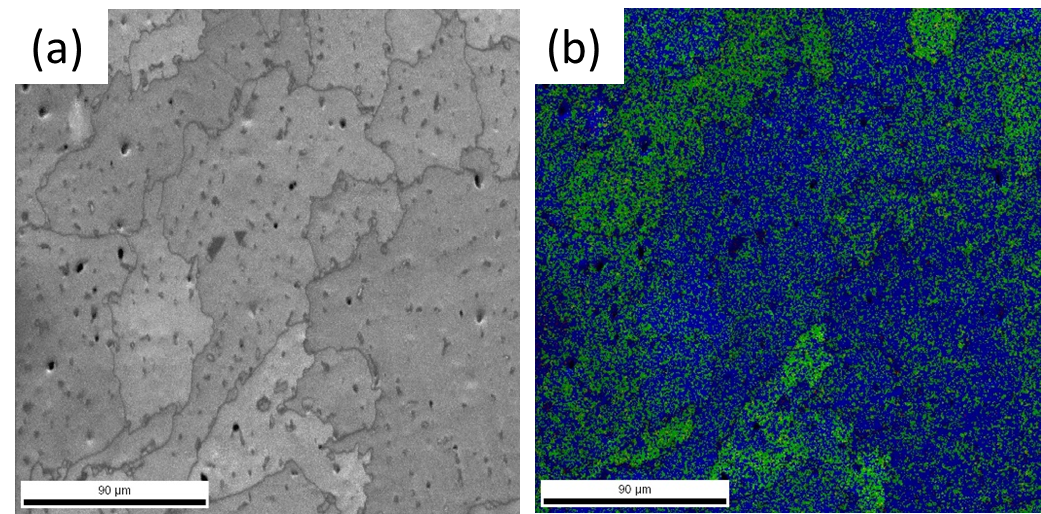


**Figure S3.** High magnification EBSD image showing (a) gray scale image (b) kernel average map, inferring a high misorientation angle at specific regions.

**Table S1:** Composition of the DED produced and VAM produced alloys

|  | Cr (at. %) | Mo (at. %) | Ta (at. %) | Ti (at. %) | V (at. %) | Fe (at. %) |
| --- | --- | --- | --- | --- | --- | --- |
| DED | 4.7 ± 0.4 | 29.7 ± 0.9 | 32.4 ± 0.9 | 12.8 ± 0.5 | 18.5 ± 0.7 | 1.8 ± 0.2 |
| VAM | 4.8 ± 0.8 | 24.6 ± 3.3 | 27.9 ± 2 | 16.2 ± 1.4 | 26.5 ± 3.2 | N/A |

**Table S2:** Microhardness values on the LP-DED produced alloys

| **Sample 1** | | |  | **Sample 2** | | |
| --- | --- | --- | --- | --- | --- | --- |
| Hardness (HV) | | |  | Hardness (HV) | | |
| Line 1 | Line 2 | Line 3 |  | Line 1 | Line 2 | Line 3 |
| 547 | 572 | 570 |  | 550 | 546 | 559 |
| 560 | 557 | 597 |  | 524 | 533 | 557 |
| 524 | 518 | 561 |  | 558 | 576 | 510 |
| 546 | 556 | 539 |  | 593 | 550 | 573 |
| 536 | 586 | 579 |  | 601 | 589 | 605 |
| 590 | 571 | 577 |  |  |  |  |
| 593 | 602 | 630 |  |  |  |  |
| 555 | 481 | 597 |  |  |  |  |
| 556±25 (Avg) | 555±39 (Avg) | 581±27 (Avg) |  | 565±32 (Avg) | 559±23 (Avg) | 561±34 (Avg) |
|  |  |  |  |  |  |  |
| **Average** | **St dev** | **Indent separation** |  | **Average** | **St dev** | **Indent separation** |
| **564** | **31.2** | **~0.5 mm** |  | **562** | **27** | **~0.5 mm** |

**Table S3:** Composition of the FCC second phase after irradiations at various conditions

|  | **Ti (at%)** | **O (at%)** | **Ta+V (at%)** | **Ta+V+Mo+Cr (at%)** |
| --- | --- | --- | --- | --- |
| 3 dpa, RT | 83 | 9 | 8 |  |
| 10 dpa, RT | 77 | 17 | 6 |  |
| 30 dpa, RT | 50 | 17 | - | 33 |
| 10 dpa, 650C | 76 | 18 | 6 |  |
| 30 dpa, 650C | 69 | 25 | 6 |  |
